# Scientific Evidence and Rationale for the Development of Curcumin and Resveratrol as Nutraceutricals for Joint Health

**DOI:** 10.3390/ijms13044202

**Published:** 2012-03-30

**Authors:** Ali Mobasheri, Yves Henrotin, Hans-Konrad Biesalski, Mehdi Shakibaei

**Affiliations:** 1 Musculoskeletal Research Group, School of Veterinary Medicine and Science, Faculty of Medicine and Health Sciences, The University of Nottingham, Sutton Bonington, LE12 5RD, UK; 2 Bone and Cartilage Research Unit, Institute of Pathology, University of Liège, Sart-Tilman, 4000 Liège, Belgium; E-Mail: yhenrotin@ulg.ac.be; 3 Department of Biological Chemistry and Nutrition, University of Hohenheim, D-70593 Stuttgart, Germany; E-Mail: Hans-K.Biesalski@uni-hohenheim.de; 4 Musculoskeletal Research Group, Institute of Anatomy, Ludwig-Maximilian-University Munich, Pettenkoferstrasse 11, D-80336 Munich, Germany; E-Mail: mehdi.shakibaei@med.uni-muenchen.de

**Keywords:** phytochemical, curcumin, resveratrol, articular cartilage, osteoarthritis, OA, rheumatoid arthritis, RA, functional food

## Abstract

Interleukin 1β (IL-1β) and tumor necrosis factor α (TNF-α) are key cytokines that drive the production of inflammatory mediators and matrix-degrading enzymes in osteoarthritis (OA). These proinflammatory cytokines bind to their respective cell surface receptors and activate inflammatory signaling pathways culminating with the activation of nuclear factor κB (NF-κB), a transcription factor that can be triggered by a host of stress-related stimuli including, excessive mechanical stress and ECM degradation products. Once activated, NF-κB regulates the expression of many cytokines, chemokines, adhesion molecules, inflammatory mediators, and several matrix-degrading enzymes. Therefore, proinflammatory cytokines, their cell surface receptors, NF-κB and downstream signaling pathways are therapeutic targets in OA. This paper critically reviews the recent literature and outlines the potential prophylactic properties of plant-derived phytochemicals such as curcumin and resveratrol for targeting NF-κB signaling and inflammation in OA to determine whether these phytochemicals can be used as functional foods.

## 1. Introduction

For the past 160 years human life expectancy has consistently increased by a quarter of a year every year [1]. The “baby boom” generation born after World War II have now reached their mid- to late 60s. In many European countries one out of every five people is aged 65 and over. According to the Organization for Economic Co-operation and Development (OECD) [2], increases in life expectancy seen over the last few decades are likely to continue in the future. It is predicted that life expectancy will continue to increase by 2.5 years each decade, meaning that the western world’s average life expectancy should reach and exceed 100 within the next 50 years [1]. The increasing human life expectancy has resulted in an increase in the prevalence of several diseases. The main four chronic diseases that the ageing population will suffer from are: arthritis, heart problems, dementia, and diabetes. The growing burden of arthritic, rheumatic and musculoskeletal diseases will place an even greater socioeconomic burden on health systems around the world as the population ages.

According to the World Health Organization (WHO) [3], orthopedic, rheumatic and musculoskeletal conditions comprise over 150 diseases and syndromes, which are usually progressive and associated with pain and disability. They can broadly be categorized as joint diseases, physical disability, spinal disorders, and conditions resulting from trauma. These conditions are leading causes of morbidity, giving rise to enormous healthcare expenditures and loss of productivity. Knowledge of the key determinants of disability in musculoskeletal conditions is critical for reducing their burden on the world’s growing population [1,4].

The United Nations, the WHO and 37 other countries have proclaimed the years 2000–2010 as the Bone and Joint Decade [3,5–7]. This global initiative is intended to improve the lives of people with musculoskeletal disorders, such as arthritis, and to advance understanding and treatment of musculoskeletal disorders through prevention, education and research. The 10 year global initiative launched by the UN urges governments around the world to start taking action to draw attention to the growing pervasiveness and impact of musculoskeletal diseases and to reduce the social and financial burdens to society. Support for this global initiative will raise awareness of musculoskeletal health, stimulate research and improve people’s quality of life.

Musculoskeletal diseases are one of the major causes of disability around the world and have been a significant reason for the development of the Bone and Joint Decade [5–8]. Rheumatoid arthritis (RA), osteoarthritis (OA), gout and lower back pain are important causes of disability-adjusted-life years in both the developed and developing world [9].

OA is one of the most common types of arthritis [10–13]. It is a major cause of pain and disability in older individuals and is expected to place a heavy burden on healthcare systems around the world as the human population ages [14]. The incidence of OA is also expected to increase with the rise in obesity and metabolic diseases associated with being overweight [14–17]. It also affects older animals and has significant consequences for companion animal mobility and welfare [18]. OA is a degenerative disease of the whole joint [13] and involves synovial inflammation and the progressive and irreversible destruction of the extracellular matrix (ECM) of articular cartilage [10,19,20]. It is also characterized by subchondral bone sclerosis, synovial hyperplasia and osteophyte (bony outgrowth) formation [21]. OA can occur in any synovial joint but symptomatic OA in humans is most common in the knee [16]. Digits of the hand and the hip are also frequently affected. In general weight-bearing joints are the worst affected. The main risk factors for OA include age, gender, genetics, obesity, and joint injury or instability [17]. Cartilage damage in OA is detected radiographically by decreases in joint space width. However, radiographic evidence is seen only after significant cartilage degradation has already taken place.

The major consequence of all forms of arthritis is joint dysfunction, disability, chronic pain, and significant morbidity. Aside from analgesics, there are currently no effective pharmacotherapies capable of restoring the structure and function of damaged synovial tissues in any form of arthritis. Consequently, there is an acute need for novel drugs and new therapies. In the following section we provide a brief overview of cartilage structure and function before discussing the molecular and cellular events that occur in a typical synovial joint in OA.

## 2. Articular Cartilage-Structure and Function

**Figure 1:**
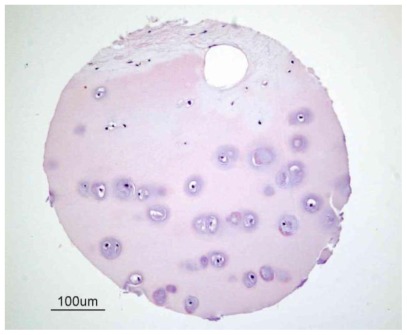
Structure of human articular cartilage. This figure illustrates a sample of human cartilage from a tissue microarray developed by the Cooperative Human Tissue Network (CHTN) [22] of the National Cancer Institute [23]. Cartilage is predominantly an avascular, aneural and alymphatic load-bearing connective tissue consisting of a single cell type known as the chondrocyte. Blood vessels are only present in subchondral bone.

Cartilage is a flexible and mechanically compliant connective tissue found at the end of long bones in articulating joints and in the intervertebral disc. It is sub-classified into three different types: elastic cartilage, hyaline cartilage and fibrocartilage, which differ in the relative amounts of its three principal components, namely collagen fibers, ground substance (proteoglycans) and elastin fibers. Articular or hyaline cartilage is a load-bearing tissue with unique biological characteristics (Figure 1).

**Figure 2:**
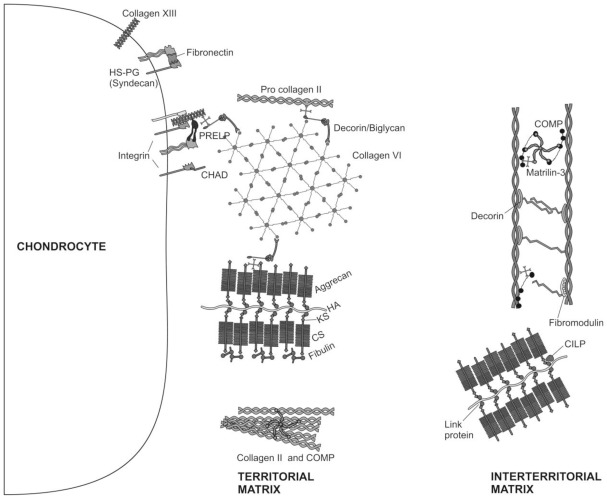
Molecular composition of the ECM of articular cartilage. The major collagenous and non-collagenous components of the territorial and interterritorial cartilage ECM are illustrated.

Its biochemical properties depend on the structural design of the tissue, the molecular composition of the ECM (Figure 2) that makes up the bulk of the tissue volume and the interactions between its resident cells and the ECM [24].

Chondrocytes are the only cells found within the cartilage ECM. Cartilage is avascular, alymphatic and aneural. Nutrition is derived from synovial fluid (and for the deep zone by subchondral bone vessels). Chondrocytes are architects of cartilage [25], building the macromolecular framework of its ECM from three distinct classes of macromolecules: collagens (predominantly type II collagens), proteoglycans (mainly aggrecan), and a variety of non-collagenous proteins (Figure 2). Of the collagens present in articular cartilage collagens type II, IX, and XI form a fibrillar meshwork that gives cartilage tensile stiffness and strength [24,26,27], whereas collagen type VI forms part of the matrix immediately surrounding the chondrocytes, enabling them to attach to the macromolecular framework of the ECM and acting as a transducer of biomechanical and biochemical signals in the articular cartilage [28,29]. Large aggregating proteoglycans (aggrecan) are embedded in the collagen mesh and give cartilage its stiffness to compression, its resilience and contribute to its long-term durability [29–32].

ECM proteins in cartilage are of great significance for the regulation of the cell behavior, proliferation, differentiation and morphogenesis [33–41]. Small proteoglycans, including decorin, biglycan, and fibromodulin are further embedded in the ECM. Decorin and fibromodulin both interact with the type II collagen fibrils in the matrix and have roles in fibrillogenesis and interfibril interactions. Biglycan is mainly found in the immediate surrounding of the chondrocytes, where it may interact with collagen type VI [24,29]. Modulation of the ECM proteins is regulated by the interaction of a diversity of growth factors with chondrocytes [42–46]. In fact, it has been reported recently, that IGF-I and TGF-β stimulate the chondrocyte surface expression of integrins, and that this event is accompanied by increasing adhesion of chondrocytes to matrix proteins [47]. Other non-collagenous proteins in articular cartilage such as cartilage oligomeric matrix protein (COMP) are less well studied and may have value as a biomarker of cartilage turnover and degeneration of [48], while tenascin and fibronectin influence interactions between the chondrocytes and the ECM [24,49]. The ECM surrounds chondrocytes; it protects them from the biomechanical stresses that occur during normal joint motion, determines the types and concentrations of molecules that reach the cells and helps to maintain the chondrocyte phenotype.

Throughout life, cartilage is continually remodeled as chondrocytes replace matrix macromolecules lost through degradation. Evidence indicates that ECM turnover depends on the ability of chondrocytes to detect alterations in the macromolecular composition and organization of the matrix, such as the presence of degraded macromolecules, and to respond by synthesizing appropriate types and amounts of new ECM components. It is known that mechanical loading of cartilage creates mechanical, electrical, and physicochemical signals that help to direct the synthesizing and degrading activity of chondrocytes [50]. In addition, the ECM acts as a signal transducer for chondrocytes [51]. A prolonged and severe decrease in the use of the joint leads to alterations in the composition of the ECM and eventually to a loss of tissue structure and its specific biomechanical properties, whereas normal physical strain stimulates the synthesizing activity of chondrocytes and possibly internal tissue remodeling [52,53].

Although articular cartilage can tolerate a tremendous amount of intensive and repetitive physical stress, it manifests a striking inability to heal even the most minor injury [52,54–56]. This makes joints particularly sensitive to degenerative processes [57]. Furthermore, aging leads to alterations in ECM composition and alters the activity of chondrocytes, including their ability to respond to a variety of stimuli such as growth factors [58–60]. All these alterations increase the probability of cartilage degeneration [55,61–63] and emphasize the importance of interaction of chondrocytes with their surrounding ECM since this interaction regulates their growth, differentiation, and survival [64].

## 3. Articular Cartilage Degradation in OA

**Figure 3:**
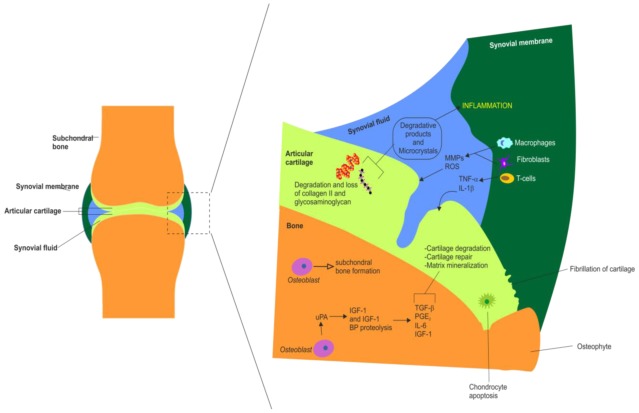
Summary of the major molecular and cellular changes that occur in the synovial joint during inflammation in OA. Summary of the major synovial, chondral and subchondral changes observed in OA. This schematic also highlights the actions of various white blood cells and inflammatory mediators in OA. Chondral changes include cartilage fragmentation (fibrillation), cartilage degradation and loss of collagen type II and glycosaminoglycans, chondrocyte apoptosis (hypocellularity) and matrix mineralization. Synovial membrane changes in OA include inflammation, synovial hypertrophy, recruitment and activation of T cells, macrophages and fibroblasts, production of matrix metalloproteinases (MMPs) and reactive oxygen species (ROS). Synovial fluid alterations in OA include accumulation of MMPs and ROS, release of IL-1β, TNF-α and other proinflammatory cytokines (IL-6, IL-8), release of inflammatory pain mediators such as prostaglandin E_2_ (PGE_2_), formation of degradative products and microcrystals. Subchondral alterations in OA include subchondral sclerosis (*i.e.*, eburnation), osteoblast mediated subchondral bone formation, proteolysis (degradation) of IGF-I and IGF-I binding proteins, increased production of some growth factors and cytokines including: transforming growth factor β, TGF-β, PGE_2_; interleukin 6, IL-6 and IGF-I.

OA (also known as osteoarthrosis or degenerative joint disease) is one of the most prevalent and chronic diseases affecting the elderly [65]. The symptoms and signs characteristic of OA in the most frequently affected joints are heat, swelling, pain, stiffness and limited mobility. OA is often a progressive and disabling disease, which occurs in the setting of a variety of risk factors, such as advancing age, obesity, and trauma, that conspire to incite a cascade of pathophysiological events within joint tissues [66]. Other sequelae include osteophyte formation and synovitis [13]. These manifestations are highly variable, depending on joint location and disease severity. Other forms of arthritis include psoriatic arthritis, and autoimmune diseases in which the body’s immune system attacks itself such as RA. Discussing these diseases in detail is beyond the scope of this review. Figure 3 outlines the major molecular and cellular changes that occur in the synovial joint in OA.

OA can affect any synovial joint but it primarily affects large load-bearing joints such as the hip and knee. The disease is essentially one acquired from daily wear and tear of the joint. Its most prominent feature is the progressive destruction of articular cartilage [11]. OA begins in articular cartilage and eventually spreads to other synovial tissues. The current consensus is that OA is a disease involving not only articular cartilage but also the synovial membrane, subchondral bone and peri-articular soft tissues [67]. OA may occur following traumatic injury to the joint, subsequent to an infection of the joint or simply as a result of aging and the mechanical stresses associated with daily life.

It is now generally accepted that OA must be viewed not only as the final common pathway for aging and injuries of the joint, but also as an active and inflammatory joint disease. As medical advances lengthen average life expectancy, OA will become an even larger public health problem—not only because it is a manifestation of aging but because it usually takes many years to reach clinical relevance. OA is already one of the ten most disabling diseases in industrialized countries. It is one of the most prevalent and chronic diseases affecting the elderly [65]. OA is rare in people under 40 but becomes more common with age—most people over 65 years of age show some radiographic evidence of OA in at least one or more joints. OA is the most frequent cause of physical disability among older adults globally. According to the National Institute of Arthritis and Musculoskeletal and Skin Diseases (NIAMS) more than 20 million Americans are estimated to have OA [68]. It is also anticipated that by the year 2030, 20% of adults will have developed OA in Western Europe and North America. Statistical data from epidemiological studies in North America and Australia suggest that arthritis is the number one condition associated with functional limitation and physical disability among US population aged 65 and older and affects 30% of the population [69]. The data from the 2003 Survey of Disability, Ageing and Carers in Australia [70] suggests that the percentage of older people with OA is even higher—around 50% profoundly and severely effecting their core activity and limiting their mobility. The reported prevalence of arthritis and its associated risk factors (*i.e.*, obesity and metabolic disease) has also increased among people aged 65 and over in nearly all European member states.

The symptoms and signs characteristic of OA in the most frequently affected joints are heat, swelling, pain, stiffness and limited mobility. OA is often a progressive and disabling disease, which occurs in the setting of a variety of risk factors, such as advancing age, obesity, and trauma, that conspire to incite a cascade of pathophysiological events within joint tissues [66]. Other important sequelae include osteophyte formation, inflammation of the synovial membrane (synovitis) and joint swelling [13]. These manifestations are highly variable, depending on joint location and disease severity. Other forms of arthritis include psoriatic arthritis, and autoimmune diseases in which the body’s immune system attacks itself such as RA. The synovitis that occurs in both the early and late phases of OA is associated with alterations in the adjacent cartilage. Catabolic and proinflammatory mediators such as cytokines, nitric oxide, prostaglandin E2 (PGE_2_) and neuropeptides are produced by the inflamed synovium and alter the balance of cartilage matrix degradation and repair, leading to excess production of the proteolytic enzymes responsible for cartilage breakdown [71]. Cartilage alterations induce further synovial inflammation, creating a vicious circle and the progressing synovitis exacerbates clinical symptoms and stimulates further joint degradation in OA [71]. Figure 3 outlines the major molecular and cellular changes that occur in the synovial joint in arthritis and synovitis.

OA, is an important cause of disability-adjusted-life years in both the developed and developing world [9]. Until recently OA was viewed as a “degenerative” or “wear-and-tear” disease and held little interest for most clinicians. It is now accepted that the age-related degeneration of articular cartilage as part of the clinical syndrome of OA is one of the most common causes of pain and disability in middle-aged and older people [9]. OA is the most common form of joint disease, with the majority of the population over 65 years of age demonstrating radiographic evidence of OA in at least one joint.

## 4. Cytokines and OA

Cytokines are signaling molecules and major mediators of inflammatory responses. They are small proteins and signaling molecules produced by a variety of different cell types. They control many different cellular functions including proliferation, differentiation and cell survival/apoptosis. They play essential an indispensable roles in cell signaling and communication and possess potent immunomodulatory properties. They are also involved in a plethora of pathophysiological processes including viral infections, autoimmune diseases, arthritis and cancer. Cytokines are synthesized under various stimuli by a variety of cells of both the innate (monocytes, macrophages, dendritic cells) and adaptive (T- and B-cells) immune systems. They have been classed as lymphokines, interleukins, and chemokines, based on their functions. The term “interleukin” was initially used by researchers for those cytokines whose presumed targets are principally leukocytes. The term “chemokine” refers to a specific class of cytokines that mediates chemo-attraction (chemotaxis) between cells. In many publications cytokines are listed along with hematopoietic growth factors, interferons, lymphokines, monokines, chemokines, and other cytokines [72]. Cytokines can be classified into two groups: proinflammatory and anti-inflammatory. Proinflammatory cytokines, including IFN-γ, IL-1β, IL-6 and TNF-α, are predominantly derived from the innate immune cells and Th1 cells. Anti-inflammatory cytokines, including IL-10, IL-4, IL-13 and IL-5, are synthesized from Th2 immune cells. The role of proinflammatory cytokines in RA is very well established [73]. Anti-cytokine therapy for RA has become a clinical treatment for aggressive forms of the disease [74]. However, proinflammatory cytokines also contribute to the pathogenesis of OA. The disease is strongly linked to aging; cell stress, injury or damage in response to chronic inflammation and exposure to cytokines, chemokines, and proteases is thought to drive its progression [75].

## 5. The Role of Cytokines in Arthritis

It is now generally accepted that proinflammatory cytokines are pleiotropic contributors to synovial joint pathology in OA and RA. In the following sections we discuss the principal cytokines involved in the pathogenesis of OA and its progression. Many studies have demonstrated the involvement of cytokines in the pathogenesis of OA (Figure 3). They are involved with synovial membrane, cartilage and bone changes in the disease process. It is now thought that much of the cytokine expression is initially by the synovium, predominantly from the synovial macrophages, which drive the inflammatory and destructive responses in OA [76]. These cytokines are thought to diffuse through the synovial fluid into the cartilage where they stimulate the chondrocytes and synoviocytes to synthesize further cytokines as well as degradative proteases. The intimal cells of the synovium are most significant in the production of cytokines that cause inflammation [77]. The main proinflammatory cytokines thought to be involved in the pathogenesis of OA are Tumor Necrosis Factor α (TNF-α) and Interleukin-1 β (IL-1β) which act on synoviocytes and chondrocytes through specific interactions with cytokine receptors on the cell surface. The receptors thought to be involved in OA are the IL-1β receptor, IL-1R type I, and the TNF-α receptor, TNF-R55, due to their elevated expression in OA human synovial fibroblasts [78,79].

There are also other proinflammatory cytokines produced by the synovium and involved in the OA disease process to a lesser extent. The effects of the proinflammatory cytokines on cartilage are shown in Table 1.

## 6. NF-κB Signaling in Arthritis

The activation of NF-κB (nuclear factor-κB) transduction pathway has been linked with a variety of inflammatory diseases, including cancer, atherosclerosis, myocardial infarction, diabetes, allergy, asthma, arthritis, Crohn’s disease, multiple sclerosis, Alzheimer’s disease, osteoporosis, psoriasis, septic shock, and AIDS [118,119]. As an activator of many pro-inflammatory cytokines and inflammatory processes NF-κB is a principal target to alleviate the symptoms of such inflammatory diseases [120]. NF-κB is a rapidly acting primary transcription factor found in all cell types. It is involved in cellular responses to proinflammatory stimuli such as cytokines and stress and plays a key role in regulating the immune response to infection. NF-κB can be triggered by a host of stress-related stimuli including proinflammatory cytokines, excessive mechanical stress and ECM degradation products [121]. In unstimulated cells NF-κB dimers are sequestered inactively in the cytoplasm by a protein complex called inhibitor of κB (IκB). IκB inactivates NF-κB by masking the nuclear localization signals (NLS). Activation of NF-κB occurs via degradation of IκB, a process that is initiated by its phosphorylation by IκB kinase (IKK). Phosphorylated IκB becomes dissociated from NF-κB, unmasking the NLS. Phosphorylation also results in IκB ubiquitination and targeting to the proteasome. NF-κB can now enter the nucleus and regulate gene expression. NF-κB turns on expression of IκB forming a negative feedback loop. Targeted strategies to prevent unwanted or excessive NF-κB activation are the focus of current research. Work in this area is focused on the use of highly specific drug modalities, siRNAs or other biological inhibitors [121]. Further work is needed to evaluate the effects of efficacious, targeted NF-κB inhibitors in animal models of OA disease *in vivo* and to also target these strategies only to affected cartilage and joints to avoid other undesirable systemic effects [121].

Recent research has shown that the pathway that activates NF-κB can be interrupted or functionally modulated by naturally occurring phytochemicals derived from spices such as curcumin, capsaicin, eugenol, gingerol, anethol, ursolic acid, diallyl sulfide, *S*-allylmercaptocysteine, ajoene, and ellagic acid [118].

## 7. Curcumin and Resveratrol—Naturally Occurring NF-κB Inhibitors

Current treatments for OA and gut are associated with unwanted side effects and are expensive. Natural products do not have such disadvantages, offer alternative treatment options for OA [122,123]. Traditional and complementary medicine is known to be fertile ground for the source of modern medicines [124]. In many different chronic diseases (including OA) in which inflammation is known to play a central role, plant derived phytochemicals (*i.e.*, curcumin and resveratrol) have been shown to exhibit therapeutic potential. The main aim of OA therapy is to counteract the local chronic inflammation, associated inflammatory symptoms in the joints, delay joint degradation, reduce and minimize disability and provide a better quality of life for patients. It is recognized that current treatments for arthritis are inefficient, cause substantial side effects, and tend to be expensive (especially when the is cost of treatment is calculated and spread over the long time course of the disease). However, natural products do not have such disadvantages and offer novel and complementary treatment opportunities [122,123]. A number of natural substances have been investigated for their anti-inflammatory capabilities, including omega-3 fatty acids (FA) [125], curcumin [126], resveratrol [127], the polyphenolic green tea catechins [128,129], and various flavonoids [130,131]. Many of them have the ability to interfere with inflammatory processes and their mediators. Thus their use along with NSAIDs may reduce inflammation and damage to joint tissues and could be of prophylactic and therapeutic value. Therefore, naturally occurring compounds capable of blocking NF-κB mediated catabolic activity may prove to be promising therapeutic agents for the treatment of OA and other inflammatory conditions. This realization has resulted in the proliferation of new research aimed at understanding how nutrients and genes interact. This new field is known as nutrigenomics and this paper’s focus on curcumin and resveratrol and many other studies in the literature highlight how these compounds target transcription factors such as NF-κB, AP-1, Egr-1, STATs, PPAR-γ, β-catenin, nrf2, EpRE, p53, CBP, and androgen receptor (AR) and AR-related cofactors [132].

## 8. Curcumin

*Curcuma longa* or turmeric is a tropical plant native to south and southeast tropical Asia. It is a member of the ginger family (Zingiberaceae) and is one of the most important of the Indian spices. Curcumin (diferuloyl methane) is the principal curcuminoid and the most active component in turmeric. It may make up 2–5% of the total spice in turmeric. Commercial curcumin contains three major components: diferuloylmethane (82%), demethoxycurcumin (15%) and bisdemethoxycurcumin (3%), together referred to as curcuminoids [133], all of which have anti-inflammatory activity. Turmeric has been used in Ayurvedic Medicine (traditional Indian medicine) for thousands of years to treat various common diseases including gastrointestinal diseases (*i.e.*, stomach ulcers), jaundice, arthritis, wounds and skin and eye infections [134–136]. Preclinical and clinical studies have shown that curcumin has potential therapeutic value against most chronic diseases including neoplastic, neurological, cardiovascular, pulmonary, metabolic and arthritic diseases. Several recent studies have also shown that curcumin has potential for the complementary treatment of arthritis [137,138].

The first preliminary study on the anti-rheumatic activity of curcumin was published in 1980. Unfortunately, this study was fundamentally flawed because of the lack of appropriate controls [139]. Studies in a rat model of joint inflammation showed that oral administration of capsaicin and curcumin lowered the levels of paw inflammation [140]. In early 2000 work on synovial fibroblasts derived from RA patients showed that curcumin inhibits the macrophage migration inhibitory factor (MIF) induced up-regulation of matrix metalloproteinases MMP-1 (interstitial collagenase) and MMP-3 (stromelysin) [141]. Curcumin strongly inhibits collagenase and stromelysin expression at micromolar concentrations [142]. Curcumin is actually a potent inhibitor of MIF [143]. Curcumin is a potent inhibitor of the production of inflammatory and catabolic mediators by chondrocytes, suggesting that this natural compound could be efficient in the treatment of OA [144]. Curcumin can induce apoptosis and inhibit prostaglandin E(2) (PGE_2_) production in synovial fibroblasts of patients with RA, suggesting that curcumin might be used to control hyperplasia of the synovial fibroblasts in RA [145].

In chondrocytes curcumin was shown to suppress oncostatin M (OSM) stimulated STAT1 phosphorylation, DNA-binding activity of STAT1, and c-Jun *N*-terminal kinase activation as well as inhibiting OSM-induced MMP-1, MMP-3, MMP-13, and TIMP-3 gene expression [146]. Curcumin was also shown to induce a 48–99% suppression of MMP-3 and 45–97% downregulation of MMP-13 in human chondrocytes and 8–100% (MMP-3) and 32–100% (MMP-13) in bovine chondrocytes [147]. Curcumin was also shown to suppress TNF-α-induced MMP-13 expression in primary chondrocytes and SW1353 chondrosarcoma cells [135].

Work from our laboratories has demonstrated some of the protective and anti-inflammatory effects of curcumin using biochemical and morphological techniques. To test the hypothesis that curcumin protects chondrocytes from morphological alterations induced by IL-1β, we investigated its *in vitro* effects on apoptotic signaling proteins and key cartilage-specific matrix components in IL-1β-stimulated chondrocytes. Transmission electron microscopy was employed to demonstrate that curcumin inhibits the early degenerative changes induced by IL-1β [148]. Additionally, curcumin antagonized the suppression of collagen type II and β1-integrin synthesis and caspase-3 activation induced by IL-1β was inhibited by curcumin [148]. This study clearly demonstrated that curcumin exerts anti-apoptotic and anti-catabolic effects on IL-1β-stimulated articular chondrocytes and may have novel therapeutic potential for treating OA and related osteoarticular diseases [148]. We used an explant model of cartilage inflammation to demonstrate that IL-1β-induced ECM degradation and glycosaminoglycan release can be inhibited by curcumin [13].

Curcumin appears to exert its anti-inflammatory effects in a “concentration-dependent” or “dose-dependent” manner. Studies on RA-derived synovial fibroblasts have shown that curcumin dose-dependently abrogates the effect of IL-18 on VEGF production [149]. Interestingly, a study on biological activities of turmeric extract has shown that the three major curcuminoids in turmeric are responsible for its anti-arthritic effects while the remaining compounds in crude turmeric extracts may actually inhibit its anti-inflammatory and protective effects [150]. Studies on other curcuma plants have shown that there is a possible curcuminoid-independent pathway mediated by *curcuma phaeocaulis* extract [151]. Further studies are required to corroborate these findings.

Treatment of chondrocytes with curcumin suppresses IL-1β-induced NF-κB activation via inhibition of IκBα phosphorylation, IκBα degradation, p65 phosphorylation and p65 nuclear translocation [152]. Curcumin also inhibits the IL-1β-induced stimulation of up-stream protein kinase B Akt, molecular events that correlate with down-regulation of NF-κB targets including COX-2 and MMP-9 [152].

Curcumin is also able to antagonize the IL-1β and TNF-α-dependent up-regulation of MMPs and COX-2. Curcumin has been shown to inhibit the inflammatory and apoptotic effects of IL-1β on chondrocytes and this correlates with down-regulation of NF-κB-specific gene products that are known to mediate inflammation, degradation and apoptosis of chondrocytes in OA. Additionally, both curcumin suppressed IL-1β-induced down regulation of the cartilage specific ECM component collagen type II and of the cartilage specific master transcription factor Sox-9. Furthermore, inhibition of NF-κB activation by curcumin occurs mainly through the IKK inhibition [135,138,141,152].

**Figure 4:**
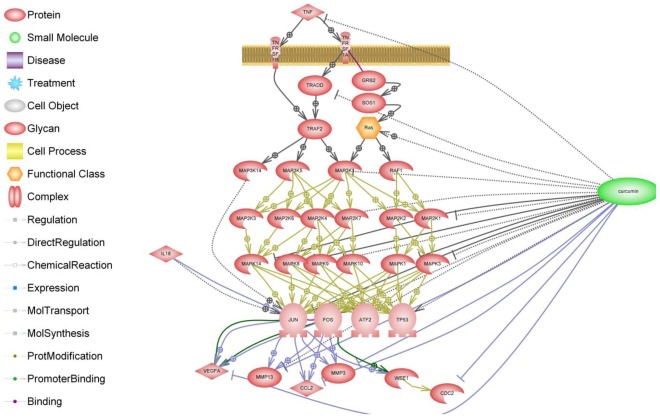
Schematic of the effects of curcumin on the TNF-α receptor and its downstream signaling pathway. The biochemical pathway illustrated here was generated by text mining and makes use of a collection of canonical Ariadne pathways in addition to MedScan text mining.

When considering the biological effects of curcumin in cartilage and synovial cells and joint tissues, the overriding question is whether curcumin is safe. The research conducted to date with curcumin suggests that it has a good safety record. However, this is not supported by clinical evidence and data from clinical trials. Another important and largely neglected issue is the bioavailability of curcumin and curcuminoids, which is poor generally. Enhancing the bioavailability of curcumin is an important and goal and is likely to bring this promising natural product to the forefront of therapeutic agents for treatment of human diseases [153]. Curcumin is also a powerful inhibitor of inflammatory pathways and mediators. The schematic shown in Figure 4 summarizes the available information in PubMed on the effects of curcumin on the TNF-α receptor and its downstream signaling pathway.

Its anti-catabolic effects, namely reducing degradative enzyme expression and activity, and its positive influence on anabolic gene expression suggests that it may be a suitable adjunct to conventional pharmaceutical (*i.e.*, NSAID) therapy. The available information suggests that curcumin could be an alternative to NSAIDs. In contrast to NSAIDs, curcumin has no gastrointestinal side effects, and can even protect the gastric mucosa. Therefore, curcumin could be beneficial in the management of chronic inflammatory-related joint disease, including OA. However, despite this optimistic view, it must be recognized that there is still a paucity of data regarding possible adverse effects of curcumin at concentrations that are biologically effective *in vitro*. Indeed, the absence of systemic adverse effects after oral administration of curcumin is probably the result of its poor bioavailability and chemical modification by the gut and liver. Whilst some evidence exists for toxicity, at super-physiological concentrations, these are unlikely to be experienced or achieved *in vivo*. Nevertheless, we cannot exclude the possibility that increasing curcumin absorption, by chemical or natural process, could have unsuspected deleterious effects. It is now documented that curcumin at concentrations in excess of 50 mM shows cytotoxicity in a chondrocyte cell line [154]. The relevance of this toxicity in *in vitro* models is highly questionable. Nevertheless, the long-term effects of curcumin have not been studied and there is no published information about the possible side effects of the metabolites of curcumin. Further work is therefore required to address the issues of bioavailability and tissue accumulation in order to calculate appropriate dose formulations to assess whether curcumin can be convincingly considered as an aid to treating OA. Curcumin regulates inflammatory cytokines such as IL-1β, IL-6, IL-12, TNF-α and IFN-γ and associated JAK-STAT, AP-1, and NF-κB signaling pathways in immune and connective tissue cells [155].

## 9. Clinical Trials of Curcumin

There are currently no registered clinical trials of curcumin in OA. “Curcumin in Rheumatoid Arthritis” (ClinicalTrials.gov [156] Identifier: NCT00752154 [157]) is a clinical trial registered on the Clinical Trials database. The study is sponsored by University of California at Los Angeles. It is a randomized, placebo-controlled crossover study in which 40 subjects will receive a total of 4 g of curcumin per day (capsule form, precise composition not disclosed) and then switch to placebo. The subjects’ participation may last up to 8 months. By completion of the study, all 40 subjects will have taken curcumin and placebo for 4 months each. Subjects will have blood tests, complete questionnaires, and be seen by the study doctor. At the present time status of this study is unknown and it looks like the original completion deadline will not be met. However, when the study is completed it will be very interesting to see if curcumin has provided any benefits for RA patients.

## 10. Bioavailability and Topical Delivery of Curcumin

The major problem associated with the use of curcumin as a drug is its low bioavailability. A recent study has attempted to enhance the bioavailability of curcumin by complexation with phosphatidyl choline followed by pharmacokinetic studies in rats [158]. The complex was shown to have significantly increased absorption compared with curcumin, when given in equimolar doses. The complex also showed enhanced bioavailability, improved pharmacokinetics, and increased hepatoprotective activity as compared with curcumin [158]. The authors have proposed that the enhanced bioavailability of the complex may be due to its amphiphilic nature, which greatly enhance the water and lipid solubility of the curcumin. This study opens up new opportunities for enhancing the absorption and bioavailability, and pharmacokinetics of curcumin. Several companies have already started selling products combining curcumin with phosphatidyl choline for improved absorption.

Another area of interest is enhancing the topical delivery of curcumin [159]. This approach is intended to increase the absorption of curcumin through skin. Combinations of cyclodextrins and alginates were used in a study by Hegge and colleagues to solubilize curcumin in aqueous vehicles intended for topical delivery [160]. The study concluded that a combination of hydroxypropyl-β-cyclodextrin and propylene glycol alginate enhances curcumin solubility and release from the vehicle [160]. These studies have demonstrated the importance of optimizing the solvent systems when utilizing cyclodextrins as drug carriers for topical treatments [159,160].

## 11. Synergistic Effects of Curcumin and NSAIDs

There is increasing interest in using curcumin in conjunction with NSAIDs to reduce the dosage of NSAIDs. Banerjee *et al*., (2003) used an adjuvant model of rat inflammation to demonstrate that curcumin and ibuprofen modulate inflammatory biomarkers such as C-reactive protein when used in combination [161]. Curcumin synergistically potentiates the growth-inhibitory and pro-apoptotic effects of the NSAID celecoxib in OA-derived synovial adherent cells [162]. This was one of the first studies to show that synergistic effects of curcumin and celecoxib may enable the use of celecoxib at lower and safer concentrations [162]. Evaluation of the effects of celecoxib and curcumin in patients with OA is ongoing in human clinical trials [163]. Synergistic action of curcumin and conventional NSAIDs is an interesting concept and may pave the way for a novel combination treatment in OA and other rheumatologic disorders [162]. Ongoing clinical trials should provide a deeper understanding of the mechanisms and therapeutic potential of curcumin [164].

## 12. Resveratrol

Resveratrol or *trans-*3,5,4′-trihydroxystibene is a polyphenolic, antifungal natural phytoalexin found in grapevines (*Vitis vinifera*) and a variety of other plants. It is found in the vines, roots, seeds and stalks, but its highest concentration is in grape skins. Resveratrol has been shown to possess potent anti-inflammatory, antioxidant and anticancer properties. It has been studied because of its anti-carcinogenic, anti-inflammatory and cardioprotective properties (coronary artery protection cumulating in the so called “French Paradox”) [165]. In addition resveratrol is thought to as suppresses angiogenesis and prevent diabetes mellitus. There are also suggestions that it may prolong lifespan [166–168]. Since resveratrol is a potent and specific inhibitor of cytokine-induced NF-κB activation, it may have potential for treating OA [137,138,143,169,170].

Studies in the rat suggest that resveratrol is absorbed in the duodenum. However, resveratrol-glucuronide was the major form absorbed when compared to the minute amounts of unconjugated resveratrol and resveratrol-sulfate [171]. Resveratrol is glucuronated in the liver and sulfated in both the liver and the duodenum [172]. The major derivatives of resveratrol glucuronidation are *trans*-resveratrol-3-*O*-glucuronide, *trans*-resveratrol-4′-*O*-glucuronide, and *trans*-resveratrol-3-*O*-sulfate [173]. Therefore, resveratrol exhibits numerous different mechanisms of action and targets are great number of intracellular molecules.

## 13. Resveratrol and Transcription factor NF-κB

As discussed earlier many inflammatory factors involved in arthritis, are regulated by the transcription factor Nuclear Factor-κB (NF-κB) [174]. NF-κB regulates many important signaling pathways in diseases with an inflammatory component [175–177]. Resveratrol blocks TNF-α-induced activation of NF-κB and suppresses TNF-α-induced phosphorylation and nuclear translocation of the p65 subunit of NF-κB and NF-κB-dependent reporter gene transcription [178].

Resveratrol is a potent inhibitor of the dioxygenase activity of lipoxygenases. Lipoxygenases are dioxygenases with peroxidase activity involved in the synthesis of mediators for inflammatory, atherosclerotic, and carcinogenic processes. Additionally resveratrol can inhibit lipoxygenases through being oxidized by their peroxidase activity. Resveratrol and its oxidized form can act as inhibitors of the dioxygenase activity of lipoxygenase [179].

## 14. Resveratrol and OA

The phenomena that inflammatory cytokines such as IL-1β and TNF-α stimulate matrix degrading enzymes such as matrix metalloproteinases (MMPs) and cyclooxygenase-2 (COX-2), through activation of NF-κB, leading to cartilage matrix destruction, joint inflammation and play an important part in pathogenesis of RA and OA [152]. COX-2 activation stimulates prostaglandin production mediating inflammation [180]. The classical treatment for OA and RA is with COX inhibitors. However, NSAIDs have well known and severe side effects such as gastric ulcerations and do not inhibit the production of inflammatory stimulating mediators. Thus, degradation of joint cartilage is further promoted. This is why there is an emerged request for anti-inflammatory treatment that on one hand inhibits COX-2 (and thus prostaglandin production) but on the other hand further block the continuing joint degeneration. Interestingly, Subbaramaiah and co-workers have demonstrated that resveratrol has COX-2 inhibitory effects. The addition of pure resveratrol inhibited COX-2 expression and the production of prostaglandin E2 [181]. Furthermore, Elmali *et al.* demonstrated that intra-articular injections of resveratrol in rabbit inflammatory arthritis model had a chondroprotective effect on the cartilage [169,182]. We have shown that resveratrol has anti-apoptotic effects on primary chondrocytes by inhibiting the IL-1β-induced stimulation of caspase-3 and the cleavage of the DNA repair enzyme poly(ADP-ribose)polymerase (PARP) in human articular chondrocytes [183]. Furthermore, we have demonstrated that resveratrol inhibits the cysteine protease caspase-3 and the subsequent cleavage of the DNA repair enzyme PARP and the IL-1β-induced up-regulation of reactive oxygen species (ROS) in chondrocytes [137].

*In vitro* studies have shown that IL-1β-induced suppression of chondrocyte proliferation and morphological alterations are suppressed by resveratrol. Resveratrol inhibits membrane-bound IL-1β and mature IL-1β protein production in chondrocytes. Furthermore, co-treatment of IL-1β-stimulated cells with resveratrol blocks activation of caspase-3, PARP cleavage, apoptosis and accumulation of tumor suppressor gene protein p53 and induces ubiquitin-independent degradation of p53. Resveratrol suppresses IL-1β-induced, NF-κB-dependent proinflammatory and matrix degrading gene products including MMPs, caspase-3, VEGF and COX-2. Resveratrol inhibits IL-1β-induced IκB-α degradation and consequently accumulated IL-1β-induced IkB-α phosphorylation. Resveratrol suppressed IL-1β-induced NF-κB dependent expression of apoptosis-related gene products by the accumulation of phosphorylated IκB-α, ubiquitinated IκB-α and inhibition of proteasome activity [137,138,183,184]. The *in vivo* effects of intra-articular injections of resveratrol on cartilage and synovium have been studied in a rabbit model of OA [169]. Resveratrol reduces cartilage tissue destruction and may protect cartilage against the development of experimentally induced OA.

There is increasing evidence to show that resveratrol may act on the sirtuin system. The silent information regulator (SIR) genes (sirtuins) comprise a highly conserved family of proteins. SirT1, the first member of the sirtuin family, is an enzyme that deacetylates proteins that contribute to cellular regulation (reaction to stressors, longevity). Two recent review articles have examined the published research on resveratrol’s effects on the expression and function of sirtuins [185]. These papers also discuss the dietary, lifestyle, and environmental factors that influence sirtuin activity, especially dietary activators like resveratrol.

## 15. Clinical Trials of Other Phytochemical Based Products Approved as Medical Foods

Limbrel [186] is a prescription medical food product for the clinical dietary management of the metabolic processes of osteoarthritis (OA). Limbrel was developed and formulated specifically for patients with OA. Although it is not a NSAID, nor a COX-2 selective inhibitor, it is proposed to function as an anti-oxidant as well as being a dual inhibitor of the cyclooxygenase (COX) and lipoxygenase (LOX) enzymes of arachidonic acid metabolism. Limbrel is manufactured according to FDA (Food and Drug Administration) current Good Manufacturing Practices (cGMP). It contains flavocoxid, a proprietary blend of natural ingredients from phytochemical food source materials. Flavocoxid is comprised primarily of the flavonoids such as baicalin and catechin. These or similar ingredients can be found in common foods such as soy, peanuts, cauliflower, kale, apples, apricots, cocoa and green tea. The fact that these and similar ingredients have been widely researched and used in medicinal products in other countries also supports biacalin and catechin’s safety and effectiveness. Limbrel provides levels of these flavonoids needed to meet the distinctive nutritional requirements of people with osteoarthritis and cannot be obtained through simply changing the diet. A recently conducted clinical trial of Limbrel (ClinicalTrials.gov [156] Identifier: NCT00928837) [187] has shown Limbrel to be effective in safely managing the unique nutritional needs of OA with side effects comparable to placebo. The Primary and Secondary Outcome Measures were to compare the efficacy, safety, quality of life and economic impact of Limbrel compared to the NSAID Naproxen and placebo.

## 16. Concluding Remarks

Nutrigenomics is an exciting area of research that holds much promise for the development of novel therapeutic strategies for the treatment of inflammatory diseases. However, wider acceptance of nutritional intervention, dietary supplements and nutraceuticals by medical practitioners arthritis patients and the scientific research community will require multi-disciplinary approaches that combine original hypothesis driven research with well-designed basic, clinical and epidemiological studies. The published data supporting the anti-inflammatory and anti-catabolic effects of curcumin and resveratrol and their synergistic activity is quite robust. Recent work has shown that curcumin and resveratrol protect chondrocytes from the catabolic actions of IL-1β including MMP-3 up-regulation, inhibition of collagen type II and down-regulation of β1-integrin expression. These phytochemicals can blocks IL-1β-induced proteoglycan degradation, AP-1/NF-κB signaling, chondrocyte apoptosis and activation of caspase-3. Therefore phytochemicals may be a beneficial complementary treatment for OA. However, more basic research is required to understand the absorption and bioavailability of these compounds and gain a deeper insight into their functional effects. In addition, the basic research needs to be followed by well-designed and conducted clinical trials that meet the current expectations of food and drug agencies in Europe and North America.

The European Food Safety Authority (EFSA) [188] based in Parma, Italy has issued new guidelines and proposed new scientific requirements for health claims related to the maintenance of joints and to the reduction of the risk of developing OA. EFSA has proposed that clinical trials of functional foods and nutraceuticals should be designed in new and innovative ways to demonstrate a “beneficial physiological effect” on healthy joints. For example, new guidelines have been introduced for the substantiation of health claims related to glucosamine alone or in combination with chondroitin sulphate and maintenance of joints [189]. According to regulation EC 1924/2006 a “beneficial physiological effect” has specific meanings for function and disease risk claims.

- For function claims: To maintain or to improve a function
- For reduction of disease risk claims: To reduce a risk factor for the development of a human disease (not reduction of the risk of the disease)—a risk factor that may serve as a predictor of development of that disease

According to these new guidelines only clinical trials designed to demonstrate a beneficial physiological effect on joints or a reduction in joint degradation in people without OA should be accepted as indicative. These guidelines present some major new challenges to the scientific and clinical communities. Furthermore, they create a number of opportunities for new types of clinical trials. Since the maintenance of a “normal joint” is considered to be a beneficial physiological effect, possible outcomes related to joint structure and function may include changes in:

- Joint space width on radiographs
- Mobility
- Stiffness
- Joint discomfort (*i.e.*, pain)

Studies performed in non-diseased (including high risk) population subgroups in which the incidence of OA is the outcome measure could be used for substantiation of health claims relating to the normal maintenance of the joint. Whilst attempting to address these requirements, we need to discriminate between food and non-food supplements. Studies dealing with “non-foods” will require a much more traditional pharmacological design compared to studies on “foods”. Clearly, addressing this issue requires new strategies and large scale clinical studies lasting several decades. Such new trials will require radical rethinking of the concept of clinical trials in the OA research community. Human studies appear to be central for substantiation of clinical data and study groups should be representative of the entire population. Hierarchy of evidence is also considered; for example interventional studies are of greater significance compared to observational studies and reproducibility of the effect much be demonstrated. In addition, demonstrating efficacy of food supplements to EFSA will also require data on tolerance and safety, specifically gastric tolerance, hepatotoxicity, renal toxicity and allergenicity. Once these important obstacles have been overcome and new clinical trials have been carried out, curcumin and resveratrol may become useful alternative adjuncts to the NSAIDs that are currently used for the treatment of OA.

## Figures and Tables

**Table 1:** Proinflammatory cytokines involved in OA.

Cytokine	Expression	Functions	References
TNF-α	Synoviocytes Chondrocytes	Increase cartilage degradation and bone resorption	[80,81]
		Inhibit glycoprotein and collagen synthesis.	[82]
		Upregulate MMP expression	[83]
		Stimulate other cells to produce proinflammatory cytokines and growth factors	[84]
		Stimulate proangiogenic factor release	[85]
		Stimulate other cells to produce chemotactic cytokines	[86,87]
		Stimulate Nitric Oxide (NO) production	[88]
		Induce chondrocyte apoptosis	[89]

IL-1β	Synoviocytes Chondrocytes Macrophages	Increase cartilage degradation and bone resorption	[80,81,90]
		Inhibit proteoglycan synthesis	[91,92]
		Upregulate MMP expression	[93]
		Production of proteolytic enzymes	[94]
		Stimulate other cells to produce proinflammatory cytokines	[77]
		Stimulate other cells to produce chemotactic cytokines	[86,87]
		Stimulate proangiogenic factor release	[85]
		Stimulate NO production	[95]
		Induce chondrocyte apoptosis	[89]

IL-6	Synoviocytes Chondrocytes osteoblasts	Inhibit proteoglycan synthesis	[96]
		Reduce chondrocyte proliferation	[96]
		Increase MMP-2 activity	[97]
		Increase aggrecanase-mediated proteoglycan catabolism	[98]

IL-8	Monocytes Synoviocytes Chondrocytes Osteoblasts	Recruits leucocytes	[99]
		Neutrophil chemoattractant	[100]
		Stimulates release of proinflammatory cytokines	[101]
		Hypertrophic differentiation and calcification of chondrocytes	[102]

IL-17	Activated T-lymphocytes	Induce NO synthesis	[103,104]
		Induce MMP synthesis	[103,104]
		Increase production of IL-1β, Il-6 and IL-8	[103,105]
		Stimulate release of proangiogenic factors	[106]

IL-18	Macrophages Synovial fibroblasts	Stimulate release of proinflammatory cytokines	[107,108]
		Stimulate angiogenesis	[109]
		Induce NO synthesis	[108]
		Synovial hyperplasia and inflammatory cell recruitment	[110]
		Induce chondrocyte apoptosis	[111]
		Reduce expression of cartilage matrix components	[111]
		Up-regulate fibronectin- a mediator of cartilage destruction	[111]

Leukaemia Inhibitory Factor (LIF)	Synovial fibroblasts Chondrocytes	Stimulate proinflammatory cytokine expression	[112,113]
		Increase pro-MMP-2 synthesis	[97]
		Increase MMP-13 synthesis and activity	[114]
		Increase cartilage resorption	[115]
		Decrease proteoglycan synthesis	[116]
		Leukocyte infiltration into synovial fluid	[117]
		Increase cartilage degradation when in combination with IL-1β and TNF-α	[115]
